# One Protein to Rule them All: Modulation of Cell Surface Receptors and Molecules by HIV Nef

**DOI:** 10.2174/157016211798842116

**Published:** 2011-10

**Authors:** Alessia Landi, Veronica Iannucci, Anouk Van Nuffel, Pieter Meuwissen, Bruno Verhasselt

**Affiliations:** Department of Clinical Chemistry, Microbiology, and Immunology, Ghent University, Belgium

**Keywords:** HIV Nef, surface receptor down-modulation, CD4, CD8, MHC-I, MHC-II, CD3, CD28, DC-SIGN, CD1, CD86, CD28, T-cell activation, viral replication, viral transmission, superinfection, immune system escape.

## Abstract

The HIV-1, HIV-2 and SIV Nef protein are known to modulate the expression of several cell surface receptors and molecules to escape the immune system, to alter T cell activation, to enhance viral replication, infectivity and transmission and overall to ensure the optimal environment for infection outcome. Consistent and continuous efforts have been made over the years to characterize the modulation of expression of each of these molecules, in the hope that a better understanding of these processes essential for HIV infection and/or pathogenesis will eventually highlight new therapeutic targets. In this article we provide an extensive review of the knowledge gained so far on this important and evolving topic.

## INTRODUCTION

Receptor down-modulation is a strategy broadly used by many viruses to escape the immune system and establish the best environment for their replication and spread [1-5]. HIV-1 seems to have evolved to master the art of receptor modulation to perfection. Infection with the virus causes the differential expression of several receptors from the surface of T cells and antigen presenting cells such as dendritic cells and macrophages. These receptors are CD4 (reviewed in [6] and [7]), CD3 [8], CXCR4 [9], CCR5 [10], CCR3 [11], CD1 [12], MHC-I [13], CD8β [14], CD80 and CD86 [15], CD28 [16] and MHC-II [17] and DC-SIGN [18], and in general their down-regulation (or up-regulation, in the case of DC-SIGN) leads to enhanced virus release, reduced superinfection and disruption of immunological responses, thus creating the perfect environment for viral replication.

The small genome of HIV-1, encoding only three structural genes and six regulatory/accessory proteins [19], necessitates optimal use of the limited coding capacity to perform all viral functions. It is therefore not surprising that multifunctional proteins arose, such as Nef that determines most of the receptor down-regulation phenomena occurring during HIV-1 infection. This protein is considered important for HIV-1 infectivity and pathogenicity. The structure, reviewed by Geyer, Fackler and Peterlin [20] in 2001, and some functions of Nef have already been clarified in part, while for other functions only hypothetical mechanistic models have been proposed. In the massive amount of literature that has been produced on Nef over the years, contradictory results on the receptor modulation mechanisms have been published, probably in part due to the use of different experimental systems, including less relevant cell lines. An example of this contradiction can be found in two very recent works [21, 22]. The use of relevant cell lines, and especially of primary cells, is key to gain relevant consistent evidence and more insights in the exact ways of action and functions of the Nef protein. Here we review the biological relevance and the importance of the different receptor expression modulation processes provoked by Nef. Our hope is that the knowledge gained until now will help to better direct the efforts in the study of these mechanisms in the future.

The down-modulation of surface receptors and molecules is considered one of the most important and conserved functions of the HIV-1 Nef protein, with relevance for pathogenicity. The down-regulation of each receptor leads to specific biological consequences and all together they contribute to create a synergy necessary for the efficient replication and infection of HIV. It is believed that the down-modulation of MHC-I, CD8β, CD4, CD1a, CD28, MHC-II and CXCR4 are fundamental to ensure an efficient immune evasion [23]. The down-regulation of CD4, one of the hallmarks of HIV-1 infection, leads to the efficient assembly of viral particles, to the disruption of CD4-based immune responses and contributes to avoid superinfection [7]. Finally, the down-regulation of CD80 and CD86 is believed to interfere with naïve T cell activation [15]. All together, these phenomena lead to a perfect environment for optimal fitness and propagation of the virus and will be discussed in detail in the following paragraphs.

## HIV-1 NEF DOWN-MODULATES CELL SURFACE RECEPTORS TO HIJACK AND ESCAPE THE IMMUNE SYSTEM

The Nef protein, produced during the early stages of infection, plays a crucial role in creating the perfect environment for viral replication and reduces the ability of the infected cells to induce an immune response, leaving them isolated from any possible immune reaction that could kill it or interfere with the virus production [6]. Here, we discuss the contribution of the down-modulation of each of these molecules to the effective viral escape from the immune system.

### CD4

CD4 is the co-receptor of the T Cell Receptor (TCR) of CD4 positive T cells and also the main receptor for HIV-1: binding with the viral envelope protein Env determines a series of conformational changes that result in the eventual fusion of the viral and cellular membranes and the entry of the virus into the host cell [24]. In the early stages of infection, Nef acts in order to eliminate the CD4 receptor from the surface. It is now clear that Nef binds to CD4 and the clathrin adaptor AP-2 and uses this interaction to recruit clathrin molecules to the membrane and therefore accelerates the endocytosis of CD4 through clathrin-coated vescicles [14, 25]. It has been shown that Nef interaction with β-COP might be important to direct the endocytosed receptors to the endosomal pathways that lead to degradation [26], a finding that has been validated recently also for CD8 and CD28 down-regulation [21]. In 2009 Bonifacino *et al. *showed, in HeLa cells stably expressing the CD4 receptor and in JM T cells, that Nef has also the ability of targeting CD4 to the Multivesicular Body Pathway (MVP) for lysosomal degradation with a separated system from surface down-modulation that involves the Endosomal Sorting Complex Required for Transport (ESCRT) [27]. This finding has revived a question ongoing for many years: are there other proteins, apart from AP-2 and β-COP, involved in the down-modulation of CD4? Which is the exact pathway? It is clear that these questions need to be answered if we want to really understand this important function of the Nef protein and its effects on HIV infection. Moreover, based on research from others and our own unpublished results, we believe that validation of all these findings should be done in primary T cells, given the variability and the difference in protein trafficking between different cell lines. In addition to Nef, also two other viral proteins act to ensure an efficient down-modulation of the receptor: Env (gp160), which retains newly synthesized CD4 molecules in the endoplasmic reticulum and Vpu, which links the gp160-bound CD4 molecules to the SCF ubiquitin ligase for degradation [28]. The CD4 co-receptor is necessary for T cell activation upon recognition of the MHC-II:antigen complex and is required for productive signaling that leads to the immune response to pathogen infection [29]. Kitchen *et al.* demonstrated in mice [30] and humans [31] that the expression of the CD4 molecule on cytotoxic lymphocytes has a functional role in antiviral response *in vivo*: CD4 on highly activated CD8+ lymphocytes serves as a chemotactic receptor for IL-16, which attracts the lymphocytes to the site of inflammation, and the binding of CD4 to MHC-II is also able to up-regulate the expression of IFN-γ and FasL. HIV might use the CD4 receptor expressed on cytotoxic T lymphocytes to enter this type of cells and immediately down-regulate it from the cell surface upon entry, dysregulating FasL induction, IFN-γ production and IL-16 binding, thus reducing the efficiency of the cytotoxic response against antigen.

CD4 down-modulation does not only have consequences on the immune response, but as the HIV receptor, viral infectivity and replication are also affected, as discussed in the following paragraphs of this review.

### Major Histocompatibility Complex, Class I (MHCI)

To escape from lysis of infected cells, HIV must evade the action of cytotoxic T lymphocytes (CTLs) which, in normal conditions, function to destroy virally infected cells. CTLs can recognize infected targets via the TCR that together with CD8, recognizes the MHC-I molecule presenting the pathogen antigen on the surface of the infected cells. Nef is known to down-modulate MHC-I from the cell surface in order to successfully avoid recognition of CTLs and thus contribute to the escape of the virus from the immune system [13]. After the discovery of this function of Nef, many studies have tried to characterize the mechanism behind it. A study of Roeth *et al.* reported that Nef first binds to the cytoplasmic tail of MHC-I early in the secretory compartment, in contrast to CD4 down-regulation which happens when CD4 is already present on the surface [32]. subsequently, the Nef-MHC-I complex recruits AP-1 using a binding site that is created when the Nef-MHC-I complex is formed and stabilized thanks to the acidic and polyproline domains of Nef [32, 33]. The formation of this complex diverts MHC-I trafficking in a way that the protein is directed to lysosomes for degradation instead of being expressed on the cell surface. In a following study by the same group, the mechanism is further characterized by the validation of the role of β-COP in the trafficking of MHC-I to the degradative compartment: knock-down of β-COP hampers both CD4 and MHC-I degradation. This suggests a model in which CD4 and MHC-I are first escorted to endosomal compartments via the interaction with AP-2 and AP-1 respectively, and then led to degradation by a common pathway involving the interaction with β-COP [26]. A recent study by the same group validated these findings and added further insights by comparing the down-modulation of the MHC-I molecule with the down-modulation of other cell surface receptors and molecules by Nef. Interestingly, the study reports that the interaction between Nef and AP-1 needed to mediate the down-regulation of CD28 and CD8β requires the tyrosine binding pocket in the μ subunit of AP-1, different from the Nef-AP1 interaction that permits down-modulation of MHC-I, which is dependent on the dileucine motif within Nef. Moreover, the role of β-COP in the degradation of internalized CD4, CD8 and MHC-I is further validated [21]. It can be speculated that Nef acts predominantly in the elimination of nascent MHC-I molecules, and not on the ones already expressed on the cell surface, because only the newly synthesized molecules would harbor viral antigens, while the ones already present on the cell-surface prior to infection wouldn’t trigger an anti-HIV CTLs response and rather inhibit NK activation.

### Major Histocompatibility Complex, Class II (MHCII)

In order to impair the host immune response to viral infections, antigen presentation in the context of MHC-II is another target for viral immune subversion. MHC-II is expressed on antigen-presenting cells (APCs) such as macrophages and dendritic cells and binds to the T cell and CD4 receptors present on T-helper lymphocytes to play a fundamental role in the immune response. Loss of functional MHC-II molecules on APCs surface hampers antigen presentation and therefore leads to an absent or defective T-helper lymphocyte-mediated immune response.

Studies in HeLa cells stably transfected with CIITA (that induces the expression of genes necessary for MHC-II presentation, i.e*. *Ii, HLA-DM, HLA-DR) determined that not only the levels of mature MHC-II on the cell surface are reduced in the presence of Nef, but also that the expression of an immature MHC-II molecule, containing the invariant chain Ii, is increased and this function appears to be specific for the Nef protein, since it is not observed during infection with Nef-deleted viruses. Mechanistic studies revealed that Nef impairs the transport of the internalized invariant chain Ii to the lysosomes, where it is normally degraded. This effect was shown to be sensitive to the phosphoinositide 3-kinase (PI3K) inhibitor* LY294002*, suggesting that Nef could stimulate PI3K activation, possibly leading to recycling of the Ii chain to the cellular surface [34-37]. Taken together these data confirm the hypothesis that Nef can hamper antigen presentation by avoiding the maturation of the MHC-II complexes. In contrast, the down-regulation of mature MHC-II from the surface is determined by a completely different pathway and requires other molecular effectors. The first insights in the molecular pathway involved, came from Chaudry *et al.*, who used both murine and human monocytic-derived cell lines but also human peripheral blood mononuclear cells [17]. They showed that despite the MHC-II down-regulation from the cellular surface the total amount of MHC-II remains constant. This must be due to an intracellular accumulation and thus demonstrates that Nef retains the internalized MHC-II molecules in the endocytic vesicles, hampering their recycling and expression on the cell surface. The internalized MHC-II molecules co-localize with markers of the endosomes and lysosomes and the process seems to be dependent on cholesterol presence in the plasma membrane, Rab5 and PI3K but independent of clathrin, dynamin and other cytoskeleton proteins involved in the Nef mediated down-regulation of other surface molecules. Taken together, these studies show that Nef induces the reduction of surface mature MHC-II expression by inducing its re-localization to the lysosomes for degradation.

A study of Schindler *et al.* validated the fact that the MHC-II down-regulation/Ii-chain up-regulation function of Nef is conserved among different strains (HIV-1 Na7, HIV-1 NL4.3, SIVmac239 and HIV-2 Ben) [36]. The conservation of this function, not only among alleles of HIV-1 Nef but also in SIV and HIV-2, suggests that it is a very important function for the virus. This study confirmed the previous results and added some important observations: these effects on MHC-II expression are observed with primary isolates from HIV-1 infected patients that show progression to AIDS, while in Long Term Non Progressors (LNTPs) these functions seems to be absent. This suggests an important role of mature MHC-II down-regulation for the progression of the disease. The study of Schindler *et al.* also determined the Nef motives involved in the process: the acidic domain (EEEE) appears to be necessary for MHC-II down-regulation but dispensable for Ii chain up-regulation while the acidic residues of the C-terminal proximal loop appear to be important for the up-regulation of the Ii chain and dispensable for MHC-II down-regulation. The dileucine motif, also important in Nef-mediated CD4 down-modulation, seems important for the up-regulation of the Ii chain while the residues Pro72 and Pro75 of the PxxP motif were important for mature MHC-II down-modulation. So far, all these functions have been detected in cell lines only: the group of Schindler *et al.* performed preliminary experiments in PBMCs but could not validate the findings obtained in cell lines [36]. Once again, most mechanisms were researched in cell lines but only some of these aspects were confirmed in part in primary cells [37, 38]: given the importance of the MHC-II molecule in the immune response and the conservation of the MHC-II down-modulation function among a wide range Nef alleles, it is of great importance to define the mechanism in primary monocytes and dendritic cells, in order to have a clearer and more relevant view on the process.

### CD8

The transmembrane glycoprotein CD8, is a co-receptor of the TCR and can be found on intestinal T cells, CD8+ T cells, thymic T cell precursors and Natural Killer cells as an αα-homodimer and on thymocytes and peripheral T cells as an αβ-heterodimer [39, 40]. The latter was reported to be strongly down-modulated by HIV-1, HIV-2 and SIVmac239 Nef in T cell precursors and CD4+T cell lines [14, 21]. Results indicate that HIV-1 Nef induces the rapid internalization of the β chain of the receptor by clathrin-mediated endocytosis, recruiting the AP-2 adaptor complex to the cell surface in order to facilitate the receptor internalization. This mechanism reveals a similarity between the CD8 and the CD4 down-modulation processes [14]. The effect on the covalently linked α chain appears much more modest compared to the β chain, suggesting CD8αβ down-modulation is mediated mainly by its effect on the β chain. Recently, Leonard *et al.* found that AP-1 recruitment to the cell surface and the interaction of Nef with β-COP are necessary for CD8β down-modulation and subsequent degradation [21]. However, they couldn’t confirm the need for AP-2 in CD8β nor CD4 down-modulation by knock-down of AP-2 α while several previous reports convincingly showed the opposite by knock-down of AP-2 μ2 [14, 25] Although there is some evidence for dysfunctional antiviral CD8 T cell responses in HIV infection (for review see reference [41]), further research has to be done on whether Nef impairs infected CD8+ T cell function. The role of CD8 molecules in the recognition of antigens in association with MHC-I molecules is very important and since CD8 positive T cells can become infected in transit through a CD4/CD8 double positive state, it is possible that Nef-dependent CD8 down-regulation provides a mechanism to avoid antigen recognition and cytotoxic responses to pathogens, therefore playing an important role in the subversion of the immune system during HIV infection.

### CD80 and CD86

CD80 and CD86 are two major co-stimulatory proteins of the B7 family, present on antigen presenting cells (APCs) such as dendritic cells (DCs) and macrophages and provide a co-stimulatory signal necessary for T cell activation and survival [42]. Different studies showed CD80 and CD86 down-regulation from the cell surface in macrophage cell lines of human and murine origin as well as in human PBL-derived macrophages after HIV-1 infection [42-44]. However, a recent study from Leonard *et al.* could not show CD80 and CD86 down-regulation in primary APCs derived from human peripheral blood mononuclear cells or in human monocytic cell lines [21]. The two-pronged mechanism involved in the Nef mediated down-regulation of CD80/CD86, described by the group of Chaudry, involves both a direct binding of Nef to CD80/CD86 to mark the molecules for Rac-mediated endocytosis and the activation of the PKC-Src-TIAM-Rac pathway to trigger Rac-mediated endocytosis. Both actions are mediated by separate Nef domains based on the evidence that a point mutation in Nef was still able to activate Src and Rac but unable to bind to the cytosolic tail of CD80/CD86 and induce internalization [44, 15]. Given the role of these two molecules, it has been speculated that Nef’s ability to reduce CD80 and CD86 surface expression in infected cells can prevent the activation of naïve T cells, necessary for an efficient recognition and elimination of the target cells. T cell activation assays showed unaffected presentation of APC endogenous antigen to both MHC-II and MHC-I restricted T cell hybridomas in the presence of Nef. This activation study was conducted in a time frame in which CD80 and CD86 appeared to be already down-regulated from the cell surface but MHC-I down-regulation still had to occur and therefore the contribution of CD80 and CD86 absence on the cells could be measured in a more precise way. The same APCs were demonstrated to be extremely poor activators of naïve primary CD4+ and CD8+ T cells, consistent with loss of co-stimulatory function mediated by Nef [15]. The results of Chaudry *et al.* [15, 44] would propose an important role for Nef-mediated loss of co-stimulatory signals during HIV infection dependent on CD80 and CD86 down-regulation. However, on the other hand the evidence gathered in primary cells by Leonard *et al.* [21] questions this hypothesis and suggests indeed a role of Nef in the loss of co-stimulatory signaling, but not necessarily through modulation of CD80 and CD86. Further experiments in primary cells are therefore needed to validate the role of CD80 and CD86 down-regulation in this context.

### CD28

The CD28 molecule is expressed on T cells and is the receptor for two other clusters of differentiation expressed in APCs, i.e*.* CD80 and CD86. It therefore plays a role together with these two proteins in efficient co-stimulation for the activation of T cells. In 2001 Swigut T. *et al*. demonstrated that Nef down-modulates CD28 from the cell surface via a mechanism similar to the one involved in CD4 down-modulation [16]. This work showed, in the Jurkat T cell line, that CD28 is removed from the cell surface by both SIV (SIVmac239) and HIV-1 (NA7) alleles via accelerated endocytosis, most likely by a mechanism that involves clathrin coated pits, with the assistance of AP-2. A recent study adds more insight in the mechanism, showing that Nef cooperates with AP-1 and β-COP to down-modulate CD28 from the cell surface [21]. Although interesting, these findings are based on SupT1 T cell line transfected with chimeras of various surface markers (CD4, CD28, CD8β, CD80, CD28, CD1d) consisting of the cytoplasmic tail of the receptor and the extracellular part of HLA-A2. It would be interesting to validate these findings in primary cells that stably and consitutively express the native receptor, in order to obtain the most relevant data.

### CD1

CD1 molecules are non-polymorphic β2-microglobulin-associated trans-membrane proteins, structurally related to MHC-I. They are capable of restricting T cells to antigens but in contrast to MHC-I, specialized in presenting lipid and glycolipid antigens [45]. Five genes coding for CD1 molecules are found in humans, and can be classified into two groups according to sequence homology: group I, consisting of CD1a, CD1b and CD1c molecules, group II consists of CD1d and homologues. Both CD1a and CD1d have been shown to be down-regulated by Nef [46, 47].

CD1a is an isoform of CD1 expressed on many professional antigen presenting cells such as B cells, activated monocytes, immature dentritic cells (iDCs) and Langerhans cells [48]. This receptor has been shown to be significantly down-modulated in iDCs and in CD1a stably transfected JY cells infected with HIV-1. The internalized CD1a molecules were found to co-localize with Nef in intracellular compartments, suggesting that the endocytosis process is Nef-mediated [12]. It is believed that CD1a is important in mucosal immunity, as it can bind and efficiently present antigens to T cells [49].

CD1d, a non polymorphic MHC class-I molecule, presents lipid antigens to Natural Killer T cells (NKT) and has been shown to be down-regulated by HIV-1 Nef in Jurkat T cells and CD4/CD1d expressing HeLa cells by accelerated endocytosis from the cell membrane and retention in the trans-Golgi network [46, 47]. It is believed that as HIV-1 reduces its visibility to MHC-I restricted T cells by down-regulating MHC-I molecules from the surface of infected cells, it also reduces its visibility to CD1d restricted NKT cells by Nef-induced down-regulation of CD1d, both cell-types being critical for controlling the virus spread in HIV-1 infection [46]. Furthermore, the down-regulation of CD1d molecules on the cell surface of infected cells could be responsible for the loss of NKT cells-mediated immune response and impaired NKT cell activation following infection [46]. The loss of CD1a instead, is believed to be responsible for the disruption of mucosal immune responses.

## MODULATION OF CD3 AND CD28 TO BALANCE THE ACTIVITY OF THE INFECTED T CELLS

The activation of CD4+ T cells requires two signals in order to happen efficiently: the interaction of the TCR-CD3 complex with the MHC class I complex presenting antigen-specific peptides and a CD28/B7-mediated co-stimulation. Efficient HIV and SIV proliferation *in vitro* requires mitogenic activation or engagement of the T cell receptor [50]; however, replication in purified T cells, PBLs or PBMCs can be measured in the absence of any exogenous stimulation. It is well established that HIV-1/2 and SIV Nef promote T cell activation in order to establish efficient infection of the target cell. Based on the measurement of IL-2 synthesis and NFAT and NFkB activity, it has been shown that HIV-1 Nef increases T cell activation induced by TCR or CD28 engagement [51-54]. Moreover, gene expression profiling revealed that Nef triggers a transcriptional program in T cells which is almost identical to that of anti-CD3 T cell activation [55] which favors HIV-1 replication by up-regulating transcription factors recruited to the LTR [56].

Induction of T cell activation is beneficial for viral infection, yet it can be detrimental for the infected T cell, since repeated stimulation of the TCR results in T cell apoptosis (a process called Activation Induced Cell Death; AICD). To protect T cells from AICD, primate lentiviruses have evolved the ability to down-regulate the TCR, which might decrease T cell activation to an optimal level, able to favor viral infection without reaching AICD. Nef alleles from HIV-2, SIVsm and SIVmac efficiently down-regulate cell surface CD3 and show impaired TCR-induced NFAT activation, a function which is lacking in HIV-1 and its SIVcpz precursor [57, 58]. Although CD3 down-regulation by Nef is not fully characterized, a direct interaction between SIVmac Nef and CD3 has been demonstrated to be surely involved in the process [59, 16].

## THE ROLE OF SURFACE RECEPTOR DOWN-MODULATION IN THE PREVENTION OF SUPER-INFECTION

HIV superinfection is deleterious for the viability of the infected cell and hence the likelihood for efficient viral replication [60, 61]. Avoidance of this phenomenon by the virus was in the beginning considered to depend solely on the down-modulation of CD4. However, this topic has generated a continuous debate over the years and more recently the discovery of the down-modulation of HIV co-receptors has highlighted that also the elimination of other molecules from the cell surface may contribute to the prevention of superinfection.

Nef has been reported to modulate the surface expression of CCR5, CCR3, which function as co-receptors especially in macrophages, T helper 2 cells and microglial cells, and CXCR4, which functions as co-receptor mainly in T cells. A study from Michel *et al.* demonstrates the surface down-regulation of these co-receptors by FACS analysis in CHO cells transfected with Nef_SF2_ [11]. Additionally the study proved that also CCR1, CCR4, CCR2, CXCR1, CCR3 and CXCR2 are down-modulated when Nef is expressed in the cell. The study has also demonstrated that the down-modulation of these chemokines receptors is a conserved function among HIV-1 (SF2, NL4-3, NA7), HIV-2 (NEP) and SIV (Mac239) alleles [11] but the actual mechanism by which HIV Nef down-regulates the surface expression of the viral co-receptors is controversial. SIV Nef is dependent on clathrin mediated endocytosis for down-modulation of CXCR4 [58], while HIV-1 Nef proteins depend on an intact acidic cluster and proline rich motif for CXCR4 internalization [11, 62]. The differential mechanism used by SIV and HIV-1 Nef may account for the differential efficiency of CXCR4 down-regulation between both Nef species. SIV Nef proteins down-modulate the surface expression of CXCR4 very efficiently, while HIV Nef proteins have a less pronounced effect on CXCR4 surface expression, with HIV-2 Nef proteins being more potent than HIV-1 [62]. Receptor mutagenesis demonstrated that the cytoplasmic tail of CCR5 and CXCR4, which is critical for ligand-mediated endocytosis, was completely dispensable for this Nef activity but mutation of the highly conserved DRY motif in the second intracellular loop of these chemokine receptors abolished the Nef-mediated down-regulation of CXCR4 [11]. Depending on the cell type and the virus tropism, the presence of CCR5, CCR3 or CXCR4 is necessary for the viral entry together with an optimal expression of CD4 on the surface of the target cell. The fact that upon its entry in the cells the virus efficiently down-regulates all these receptors present on the cell surface suggests that this might happen in order to prevent the entry of other viral particles: an accumulation of proviral DNA in fact would likely cause toxicity and cell death, a condition that is not desirable for the virus to efficiently fulfill its life cycle.

Similar to other viruses [63], the importance of down-modulation of the primary receptor by HIV is a controversial topic. There are studies that support this theory, for instance it has been shown that HIV-1 infected cells, as well as SIV-Nef expressing cells, are resistant to HIV-2 superinfection. However, questions have been raised, mainly due to the kinetics of the down-regulation: in T cells, this process starts only two days after infection, shortly before cells are committed to die. This means that there is only a short time span in which CD4 down-modulation would confer resistance to superinfection. Juan Lama in his extensive review [6] proposes that, due to these kinetic observations, even if superinfection occurs, it is unlikely that this event would affect the final output of viral production and gives arguments concerning viral selection and evolutionary reasons that would justify superinfection observed in a part of HIV-infected individuals. 

We think that, although there are good arguments to question the exclusive importance of CD4 down-modulation in the avoidance of superinfection, the experimental evidences available today are not enough to support a theory in which superinfection is considered beneficial for the virus in terms of evolution and fitness, as hypothesized by Lama in his review [6]. The effort that HIV puts in down-modulating not only the CD4 receptor but also the CCR5, CCR3 and CXCR4 co-receptors and the subsequent effects on superinfection illustrates that the virus does care to avoid it. Michel *et al. *provide a very good characterization of the effect of receptor down-modulation on superinfection [10]. In this study, TZM cells expressing wild type Nef or Nef mutants defective for CD4 down-modulation were infected with HIV-1 R5 YU-2. The cells not expressing Nef were productively infected, while the ones containing Nef were almost totally protected from infection. Interestingly a Nef EDAA mutant, lacking the CD4 down-regulation function, was still showing residual protection from superinfection because it retained the CCR5 down-modulation activity. Indeed, a collaborative effect of the down-modulation of the CD4 receptor and CCR5 co-receptor is suggested by the fact that also cells expressing a Nef mutant lacking CCR5 down-modulation are protected from HIV infection, although to a smaller extent than cells expressing native Nef [10]. The authors of this study propose that the avoidance of superinfection, necessary for the effective virus replication, coexists with the need for a certain level of superinfection, necessary for an optimal recombination, selection and thus evolution of the virus. Primate lentiviruses appear to have evolved time frames during which permission or avoidance of superinfection is regulated by gene expression: only immediately after infection, but before HIV gene expression starts, superinfection could readily occur to allow generation of recombinants. A more recent study has demonstrated that Nef synthesized from unintegrated viral DNA is able to down-modulate the expression of the HIV co-receptors CXCR4 and CCR5 [64] in addition to CD4, highlighting the possibility that soon after infection Nef is produced at functionally relevant levels to induce the down-regulation of the cell receptor used for viral entry. It can be speculated that the purpose of this action is to prevent superinfection from the very early stages of infection, but additional superinfection experiments, especially on primary cells, are needed in order to confirm this hypothesis.

## THE ROLE OF SURFACE RECEPTOR MODULATION IN VIRAL REPLICATION AND VIRUS SPREAD

It is widely known and accepted that Nef can enhance viral replication through its CD4 down-modulation function. Here we will briefly review the mechanism involved in this process and the contribution of the up-regulation of the DC-SIGN molecule to viral spread. 

Among the first relevant results showing the effect of CD4 down-modulation on viral replication in CD4+ primary T cells is a study from Lundquist *et al.* [65], in which T cells were infected with an array of HIV-1 viruses containing point mutations to Nef residues previously shown to selectively impair each of Nef known activities [65]. Interestingly, the six Nef point mutants that exhibited the strongest impairment in CD4 down-regulation were also the ones inducing most delayed replication. Later on, a study of Pham *et al.* [66] evaluated the infection of various cell lines and primary cells expressing truncated CD4 molecules resistant to down-modulation: the infection proceeded normally and virions were released in normal amounts, however the infectivity of progeny virions was reduced of around 1000-fold [66]. Further studies of Lundquist *et al.* [67] determined that Nef seems to enhance infectivity and replication of HIV-1 by the stabilization of gp120 and gp41 at the cell surface. Moreover, the conclusions of this study led to the hypothesis that the removal of CD4 from the membrane would stabilize gp120 into the nascent virions while leaving them free of CD4 molecules. This possibility was already speculated by Lama *et al*. [68, 6] but the study of Lundquist provides relevant characterization and validation as it is performed in primary blood T lymphocytes.

The type II trans-membrane lectin DC-SIGN is expressed at the surface of both immature and mature dendritic cells (DCs). The function of this molecule is to mediate the transmigration of DCs through the vascular and lymphoid endothelium (via the binding with the ICAM-2 ligand) and the adhesion of DCs to T cells in the antigen presentation process to the TCR (via the ICAM-3 ligand present on the surface of T cells). In the context of HIV infection, cells expressing DC-SIGN are able to retain attached virions in an infectious state for several days and transmit them to T cells [69]. A study of Sol-Foulon *et al.* demonstrated that Nef up-regulates the levels of DC-SIGN on the surface of DCs by inhibiting its endocytosis, and that this stabilization of the lectin on the cell membrane dramatically increases the ability of DCs to cluster with lymphocytes and transmit the virus [18]. In order to determine how this happens, HeLa cells expressing Nef were used. Nef expression was shown to increase DC-SIGN levels on the cell surface and mutational analysis on the viral protein revealed that mutation of the dileucine motif in the C-terminus of Nef, also crucial for CD4 down-modulation, abolished the effects on DC-SIGN. In contrast, the mutation of the polyproline motif, important for the interaction with proteins carrying the SH3 domain and necessary for MHC-I down-modulation, did not impair the ability of Nef to modulate surface levels of DC-SIGN. The study also evaluates the functional consequences of the up-regulation of DC-SIGN in HIV infected cells. Microscopic analysis revealed a dramatic increase in the formation of clusters between HIV wild type infected DCs and activated primary T cells, while cells infected with Nef deleted viruses showed a pattern of clustering much more similar to that of non infected cells [18].

These results help to clarify an additional receptor modulation function of Nef, which seems to be important for the Nef-mediated increase of viral spread and transmission to T cells. It can be speculated that the enhanced DC-T cell clustering due to DC-SIGN up-regulation might have effects on the activation of T cells, thus contributing to the state of hyper-activation typical of HIV infection. The validation of this hypothesis by experimental evidence would add another mechanism to the already complex array of functions through which HIV Nef manipulates the immune system for the benefit of the virus. 

## CONCLUSIONS

The modulation of surface receptors by the HIV Nef protein creates a synergy necessary for the efficient replication, infection and pathogenesis of HIV.

Several studies over the years have investigated the mechanisms underlying these processes and partially characterized the protein-protein interactions involved. The efforts made up to date permit to gain insight in many of Nef receptor-modulating activities. However further studies in primary cells are needed to validate the state of the art of knowledge, since often results obtained in different cell lines have been shown to be contradictory. A better and physiologically relevant comprehension of the modulation mechanism of each receptor, crucial for optimal outcome of HIV infection, will hopefully lead to the design of new therapeutic targets and strategies.

## Figures and Tables

**Table 1.** Cell Surface Molecules Modulated by Nef

Cell Surface Receptor	Expression	Function	Down-Modulation Consequences
**CD1a**	Antigen presenting cells (dendritic cells, monocytes, Langerhans cells, B cells)	Presents antigens to T cells	Impaired immune response
**CD1d**	Antigen presenting cells (dendritic cells, monocytes, Langerhans cells, B cells)	Presents antigens to NKT cells	Impaired immune response
CD3^*^	Thymocytes, T lymphocytes	Signal transduction together with CD28 and TCR	Lowers T cell activation to an optimal balance between viral replication and cellular survival
**CD4**	CD4 + T lymphocytes monocytes, dendritic cells, NK cells	HIV-1, HIV-2, SIV receptor, immune response to pathogens, binds to MHC-II (T cell activation upon antigen recognition)	Increased replication, impaired superinfection, impaired T helper-mediated immune response
**CD8β**	CD8+ T lymphocytes, NK cells, thymocytes	Immune response to pathogen infection Binds to MHC-I (T cell activation upon antigen recognition)	Impaired cytotoxic immune response
**CD28**	T lymphocytes, NK cells	Binds to CD80 and CD86 to provide efficient co-stimulation	Impaired T cell activation Impaired immune response
**CD80/CD86**	Antigen presenting cells (dendritic cells, monocytes)	Co-stimulatory proteins of the B7 family. Provide signals for T cell activation and survival	Impaired T cell activation Impaired immune response
**CCR5**	T lymphocytes, dendritic cells, NK cells, monocytes	Chemokine receptor (RANTES, CCL3, CCL4) Co-receptor for HIV	Impaired superinfection
**CCR3**	Thymocytes, T lymphocytes	Chemokine receptor (RANTES, CCL7, CCL11, CCL26) Co-receptor for HIV	Impaired superinfection
**CXCR4**	NK cells, dendritic cells, monocytes thymocytes, T lymphocytes	Chemokine receptor (CXCL12/SDF-1) Co-receptor for HIV	Impaired superinfection
**MHC-I**	NK cells, dendritic cells, monocytes, B cells, thymocytes, T lymphocytes	Triggers cytotoxic immune response by presenting antigens to CD8+ lymphocytes	Impaired cytotoxic immune response
**MHC-II**	NK cells, dendritic cells, monocytes B cells, thymocytes, T lymphocytes	Triggers immune response by presenting antigens to CD4+ lymphocytes	Impaired T helper cell-mediated immune response
**Cell surface receptor**	**Expression**	**Function**	**Up-modulation consequences**
**DC-SIGN**	Dendritic cells	Trans-migration of DCs through vascular endothelium. Adhesion of DCs to T-cells during antigen presentation	Increased adhesion of DCs to T cells and virus transmission

**Legend:** Table shows, for every cell-surface molecule modulated by Nef discussed in the text, the typical pattern of expression on cells of the immune system, the function in normal conditions and the consequences of cell-surface expression alteration.

* Down-regulated by the Nef protein of HIV-2 and some SIV strains.
